# Development of Fibroblast Activation Protein–Targeted Radiotracers with Improved Tumor Retention

**DOI:** 10.2967/jnumed.118.224469

**Published:** 2019-10

**Authors:** Anastasia Loktev, Thomas Lindner, Eva-Maria Burger, Annette Altmann, Frederik Giesel, Clemens Kratochwil, Jürgen Debus, Frederik Marmé, Dirk Jäger, Walter Mier, Uwe Haberkorn

**Affiliations:** 1 Department of Nuclear Medicine, University Hospital Heidelberg, Heidelberg, Germany; 2 Clinical Cooperation Unit Nuclear Medicine, German Cancer Research Center, Heidelberg, Germany; 3 Department of Radiation Oncology, University Hospital Heidelberg, Heidelberg, Germany; 4 Clinical Cooperation Unit Radiation Oncology, German Cancer Research Center, Heidelberg, Germany; 5 Department of Gynecologic Oncology, National Center for Tumor Diseases and Department of Obstetrics and Gynecology, University Women’s Clinic, University Hospital Heidelberg, Heidelberg, Germany; 6 Department of Medical Oncology, National Center for Tumor Diseases, Heidelberg, Germany; and; 7 Translational Lung Research Center Heidelberg, German Center for Lung Research, Heidelberg, Germany

**Keywords:** fibroblast activation protein, PET/CT, theranostics, FAP inhibitor, tracer development

## Abstract

Cancer-associated fibroblasts constitute a vital subpopulation of the tumor stroma and are present in more than 90% of epithelial carcinomas. The overexpression of the serine protease fibroblast activation protein (FAP) allows a selective targeting of a variety of tumors by inhibitor-based radiopharmaceuticals (FAPIs). Of these compounds, FAPI-04 has been recently introduced as a theranostic radiotracer and demonstrated high uptake into different FAP-positive tumors in cancer patients. To enable the delivery of higher doses, thereby improving the outcome of a therapeutic application, several FAPI variants were designed to further increase tumor uptake and retention of these tracers. **Methods:** Novel quinoline-based radiotracers were synthesized by organic chemistry and evaluated in radioligand binding assays using FAP-expressing HT-1080 cells. Depending on their in vitro performance, small-animal PET imaging and biodistribution studies were performed on HT-1080-FAP tumor–bearing mice. The most promising compounds were used for clinical PET imaging in 8 cancer patients. **Results:** Compared with FAPI-04, 11 of 15 FAPI derivatives showed improved FAP binding in vitro. Of these, 7 compounds demonstrated increased tumor uptake in tumor-bearing mice. Moreover, tumor–to–normal-organ ratios were improved for most of the compounds, resulting in images with higher contrast. Notably two of the radiotracers, FAPI-21 and -46, displayed substantially improved ratios of tumor to blood, liver, muscle, and intestinal uptake. A first diagnostic application in cancer patients revealed high intratumoral uptake of both radiotracers already 10 min after administration but a higher uptake in oral mucosa, salivary glands, and thyroid for FAPI-21. **Conclusion:** Chemical modification of the FAPI framework enabled enhanced FAP binding and improved pharmacokinetics in most of the derivatives, resulting in high-contrast images. Moreover, higher doses of radioactivity can be delivered while minimizing damage to healthy tissue, which may improve therapeutic outcome.

Fibroblast activation protein (FAP), a member of the serine protease family, is expressed in the microenvironment of more than 90% of epithelial tumors, including pancreas, colon, breast, and ENT (ear, nose, and throat) carcinomas (1). Despite its controversial pathophysiologic role in tumor progression, overexpression of the membrane protein is associated with a poor prognosis and a fast progression of disease (2–4). On this account, FAP indisputably represents an interesting target structure for imaging and the targeted delivery of therapeutically active compounds (1,5–9). In our previous work, we presented the development of several quinoline-based theranostic radiotracers, which were successfully used for tumor imaging of a multitude of different cancers, including pancreas, breast, and colon carcinoma, as well as high-grade glioblastoma (10,11).

Originating from the initial lead structure FAPI-02, a first improvement with regard to tumor retention was already obtained by chemical modification of the molecule. Although the tumor uptake from 1 to 3 h after injection decreased by 75% for FAPI-02, tumor retention was slightly prolonged with FAPI-04 (50% washout). A comparison with the commonly used radiotracer ^18^F‐FDG revealed equal or improved tumor-to-background contrast ratios for FAPI-04 in 6 cancer patients (12). Moreover, a first therapeutic approach using β-emitting radionuclides was adopted, proving safety and harmlessness of the novel pharmaceuticals. Efficient endoradiotherapeutic use of the FAPI tracers, however, is still limited by their relatively short tumor retention time. We therefore aimed for further development of these FAP-targeting molecules to increase the total tumor dose while maintaining low unspecific binding to healthy tissue.

## MATERIALS AND METHODS

### Chemistry

All solvents and nonradioactive reagents (except for solid-phase peptide synthesis) were obtained in reagent grade from ABCR, Sigma-Aldrich, Acros Organics, or VWR and were used without further purification. All FAPI derivatives up to FAPI-36 were synthesized as previously described (10,11), whereas the attachment of the bicyclic diamines required higher temperatures and longer reaction times. The triazole ring of FAPI-37 was formed by a copper-catalyzed Huisgen reaction of an azide-substituted quinoline-4-carboxylic acid with propargylamine. For FAPI-39, -40, -41, -46, -53, and -55, a palladium-catalyzed coupling reaction was performed with *tert*-butyl 6-bromoquinoline-4-carboxylate and the individual linker reagent. More detailed information on compound chemistry and synthesis can be found in the supplemental materials (available at http://jnm.snmjournals.org).

### Radiolabeling

^177^Lu and ^68^Ga were chelated after pH adjustment with sodium acetate. The reaction mixture was heated to 95°C for 10 min, and the completeness of the reaction was checked by radio–high-performance liquid chromatography. ^177^Lu-labeled FAPIs were used directly for in vitro studies or diluted with 0.9% saline and directly applied for organ distribution studies. The ^68^Ga compounds were processed by solid-phase extraction before PET imaging.

### Cell Culture

HT-1080 cells transfected with the human FAP gene, as well as murine FAP- and CD26-transfected human embryonic kidney cells (obtained from Stefan Bauer, NCT Heidelberg (13)), were cultivated in Dulbecco modified Eagle medium containing 10% fetal calf serum at 37°C/5% carbon dioxide.

For radioligand binding studies, cells were seeded in 6-well plates and cultivated for 48 h to a final confluence of about 80%–90% (1.2–2 million cells per well). The medium was replaced by 1 mL of fresh medium without fetal calf serum. The radiolabeled compound was added to the cell culture and incubated for different intervals ranging from 10 min to 24 h. Competition experiments were performed by simultaneous exposure to unlabeled (10^−5^ to 10^−10^ M) and radiolabeled compound for 60 min. Cell efflux was determined after incubation of the cells with the tracer for 60 min. Thereafter, the radioactive medium was removed, and the cells were washed and incubated with nonradioactive medium for 1, 2, 4, and 24 h. In all experiments, the cells were washed twice with 1 mL of phosphate-buffered saline, pH 7.4, and subsequently lysed with 1.4 mL of lysis buffer (0.3 M NaOH, 0.2% sodium dodecyl sulfate). Radioactivity was determined in a γ-counter (Cobra II; Packard), normalized to 1 million cells, and calculated as percentage applied dose. Each experiment was performed 3 times, and 3 repetitions per independent experiment were acquired.

### Animal Studies

For in vivo experiments, 8-wk-old BALB/c *nu/nu* mice (Charles River) were subcutaneously inoculated into the right trunk with 5 million HT-1080-FAP cells. When the size of the tumor reached approximately 1 cm^3^, the radiolabeled compound was injected via the tail vein (80 nmol/GBq for small-animal PET imaging; 200 nmol/GBq for organ distribution). In vivo blocking experiments were performed by adding 30 nmol of unlabeled FAPI to the radiolabeled compound directly before injection. For organ distribution, the animals (*n* = 3 for each time point) were killed 1, 4, 6, and 24 h after tracer administration. The distributed radioactivity was measured in all dissected organs and in blood using a γ-counter (Cobra Autogamma; Packard). The values are expressed as percentage injected dose per gram of tissue (%ID/g). PET imaging was performed using a small-animal PET scanner (Inveon; Siemens). Within the first 60 min, a dynamic scan was performed in list mode, followed by a static scan from 120 to 140 min after injection. Images were reconstructed iteratively using the 3-dimensional ordered-subset expectation maximization maximum a priori method (Siemens) and were converted to SUV images. For the dynamic data, 28 frames were reconstructed: 4 × 5 s, 4 × 10 s, 4 × 20 s, 4 × 60 s, 4 × 120 s, 6 × 300 s, and 2 × 470 s. Quantification was done using a region-of-interest technique and expressed as SUV. All animal experiments were conducted in compliance with the German animal protection laws (approval 35-91185.81/G-158/15).

### Clinical PET/CT Imaging

Imaging of 8 patients was performed under the conditions of the updated Declaration of Helsinki, section 37 (unproven interventions in clinical practice) and in accordance with the German Pharmaceuticals Law, section 13 (2b), for medical reasons using ^68^Ga-FAPI-21 and -46, which were applied intravenously (20 nmol, 210–267 MBq for FAPI-21 and 216–242 MBq for FAPI-46). Imaging took place at 10 min, 1 h, and 3 h after tracer administration. The PET/CT scans were obtained with a Biograph mCT Flow PET/CT scanner (Siemens Medical Solutions) using the following parameters: slice thickness of 5 mm, increment of 3–4 mm, soft-tissue reconstruction kernel, and CARE Dose. Immediately after CT scanning, a whole-body PET scan was acquired in 3 dimensions (matrix, 200 × 200) in FlowMotion with 0.7 cm/min. The emission data were corrected for randoms, scatter, and decay. Reconstruction was conducted with an ordered-subset expectation maximization algorithm with 2 iterations and 21 subsets and Gauss-filtered to a transaxial resolution of 5 mm in full width at half maximum. Attenuation was corrected using the low-dose nonenhanced CT data. SUVs were quantitatively assessed using a region-of-interest technique. The data were analyzed retrospectively with approval of the local ethics committee (approval S016/2018).

### Statistical Analysis

Statistical analysis of the cell culture and animal experiments was performed using Prism 7.0 (GraphPad Software). Unless stated otherwise, all values are expressed as mean ± SD. For normally distributed populations, the means of different groups were compared using an unpaired *t* test.

## RESULTS

### Serum Stability

Processed and solvent-free radioactive compounds (^177^Lu-FAPI-21 and ^177^Lu-FAPI-46) were incubated in human sera at 37°C. After the respective incubation time, samples were taken, freed from proteins by precipitation with acetonitrile, and centrifuged, and the supernatant was analyzed via radio–high-performance liquid chromatography. Supplemental Figure 1 shows that even at 24 h, only the initial (radioactive) peaks were detected and neither radioactive degradation products nor free radioactivity were observed. These findings demonstrate that both substances were unhampered by enzymatic components of human sera.

### Chemical Modification of the FAPI Framework Resulting in Increased FAP Binding In Vitro

**FIGURE 1.**
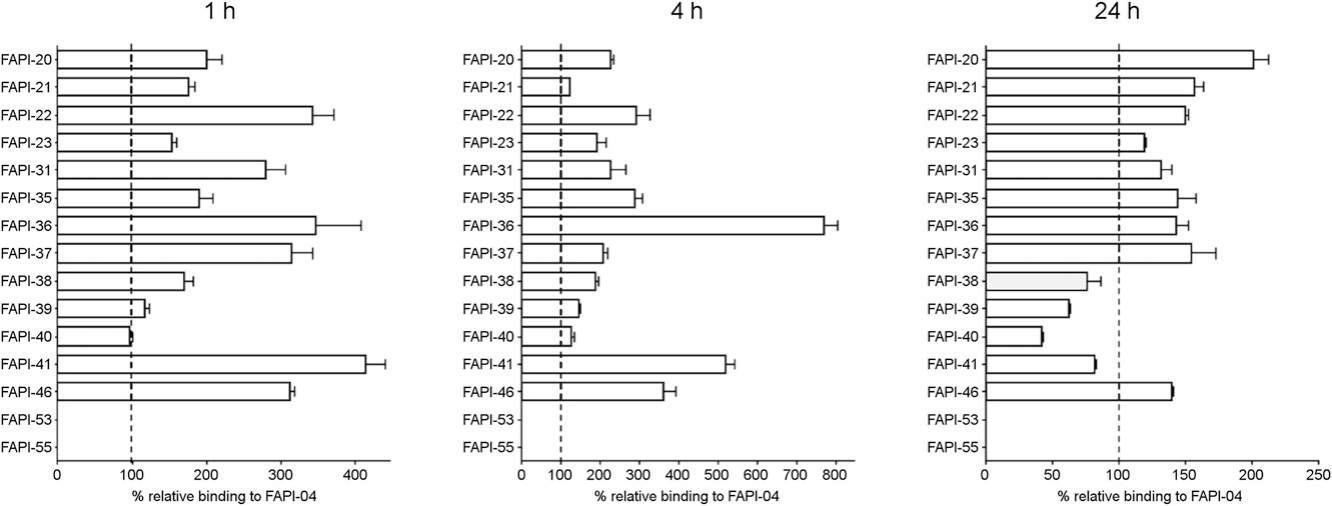
Relative binding rates of ^177^Lu-labeled FAPI derivatives compared with FAPI-04 (set to 100%) using FAP-expressing HT-1080 cells (*n* = 3).

To determine the FAP-binding affinities of the radiotracers (Supplemental Table 1), radioligand binding assays were performed using human FAP-expressing HT-1080 cells. To compensate for varying rates of FAP expression and allow a direct comparison with the lead structure, all experiments were conducted in parallel with FAPI-04. All compounds demonstrated robust binding to human FAP, with binding values equal to or higher than those of FAPI-04 after 1 and 4 h of incubation (Fig. 1). Internalization rates were comparable to those of FAPI-04 for all compounds except for FAPI-38 (63.1% internalized after 24 h; FAPI-04, 97.1%; Supplemental Table 2). Although most derivatives revealed higher binding values after 24 h than for FAPI-04, the compounds FAPI-38, -39, -40, and -41 were eliminated significantly faster from FAP-expressing cells and were, therefore, not considered for a more detailed characterization. Similar to FAPI-04, all compounds demonstrated negligibly low binding to the structurally related membrane protein CD26 (data not shown).

### Improvement of Pharmacokinetics and Image Contrast for PET

**FIGURE 2.**
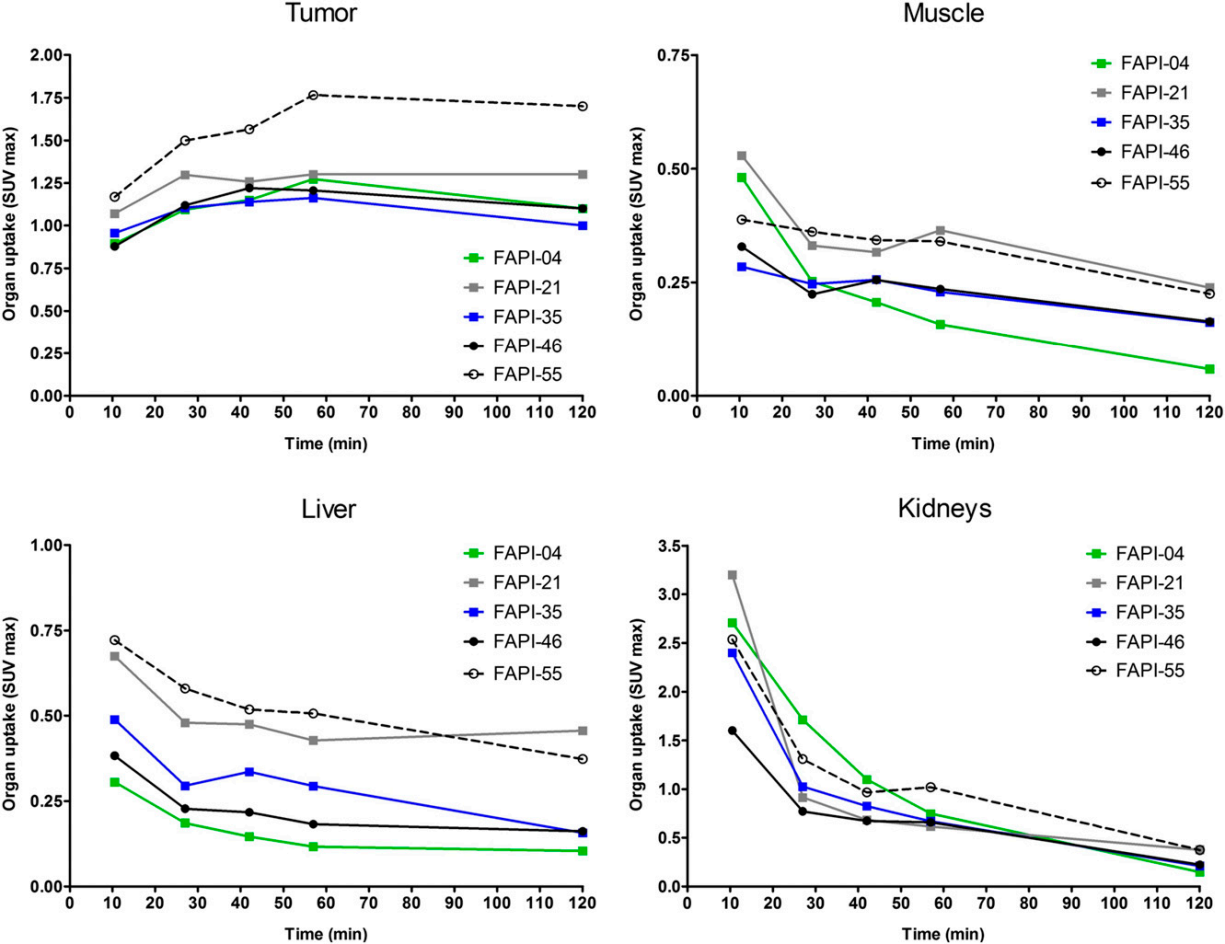
Organ SUV_max_ of ^68^Ga-labeled FAPI derivatives in HT-1080-FAP tumor–bearing mice determined by small-animal PET imaging (*n* = 1).

**FIGURE 3.**
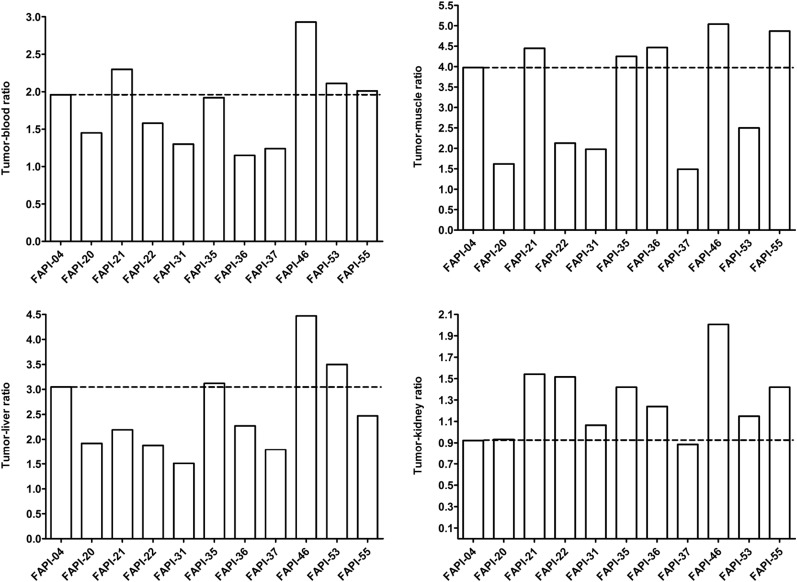
Tumor–to–normal-organ ratios of ^68^Ga-labeled FAPI derivatives, calculated from AUCs 0–2 h after intravenous administration of radiotracers (*n* = 1).

To assess a potential increase in tumor retention and to evaluate their pharmacokinetic behavior, the most promising candidates were analyzed in vivo. To this end, small-animal PET imaging was performed on HT-1080-FAP xenografted mice. All compounds demonstrated rapid tumor accumulation with overall low background activity and predominantly renal elimination (Supplemental Fig. 4). Tumor uptake was highest for FAPI-55 (SUV_max_ of 1.8 after 60 min and 1.7 after 120 min), followed by FAPI-36 (1.5 after 60 min and 1.3 after 120 min) and FAPI-21 (1.3 after both 60 and 120 min) (Fig. 2, Supplemental Fig. 5). Because the absolute uptake values allow only a limited comparison of the radiotracers, AUCs were calculated from the time–activity curves, representing the accumulated radioactivity within the interval up to 2 h after injection. As shown in Table 1, 7 of 10 compounds demonstrated higher tumor uptake than that for FAPI-04, headed by FAPI-21, -36, -46, and -55. Yet, FAPI-36 showed a prolonged systemic circulation, resulting in unfavorable tumor-to-blood ratios and a poorer image contrast than that for FAPI-04 (Supplemental Fig. 4). Although the tumor-to-blood and tumor-to-liver ratios for FAPI-35 were comparable to those for FAPI-04, the tumor-to-muscle ratio was slightly improved (Fig. 3). FAPI-21 and -55 demonstrated higher accumulation in liver and muscle tissue than did FAPI-04. From all tested compounds, FAPI-46 displayed the highest tumor-to-blood, tumor-to-muscle, and tumor-to-liver ratios.

**TABLE 1:** AUCs* and Tumor–to–Normal-Organ Ratios^†^ for ^68^Ga-Labeled FAPI Derivatives

Derivative	AUC*					Ratio^†^		
	Tumor	Blood	Kidney	Liver	Muscle	Tumor-to-blood	Tumor-to-muscle	Tumor-to-liver
FAPI-04	58.02	29.54	62.84	19.02	14.57	1.96	3.98	3.05
FAPI-20	57.75	39.71	61.92	30.08	35.56	1.45	1.62	1.92
FAPI-21	92.59	40.19	60.01	42.26	20.82	2.30	4.45	2.19
FAPI-22	63.95	40.36	42.18	33.99	30.06	1.58	2.13	1.88
FAPI-31	51.76	39.76	48.53	34.32	26.15	1.30	1.98	1.51
FAPI-35	68.11	35.56	47.96	21.83	16.01	1.92	4.25	3.12
FAPI-36	86.74	75.35	69.92	38.29	19.39	1.15	4.47	2.27
FAPI-37	50.82	41.14	57.38	28.40	34.11	1.24	1.49	1.79
FAPI-46	79.63	27.22	39.67	17.82	15.80	2.93	5.04	4.47
FAPI-53	60.85	28.80	52.91	17.40	24.30	2.11	2.50	3.50
FAPI-55	106.20	52.78	74.75	42.99	21.81	2.01	4.87	2.47

* Calculated from SUV_mean_ 0–2 h after intravenous administration.

† Calculated from AUC 0–2 h.

**FIGURE 4.**
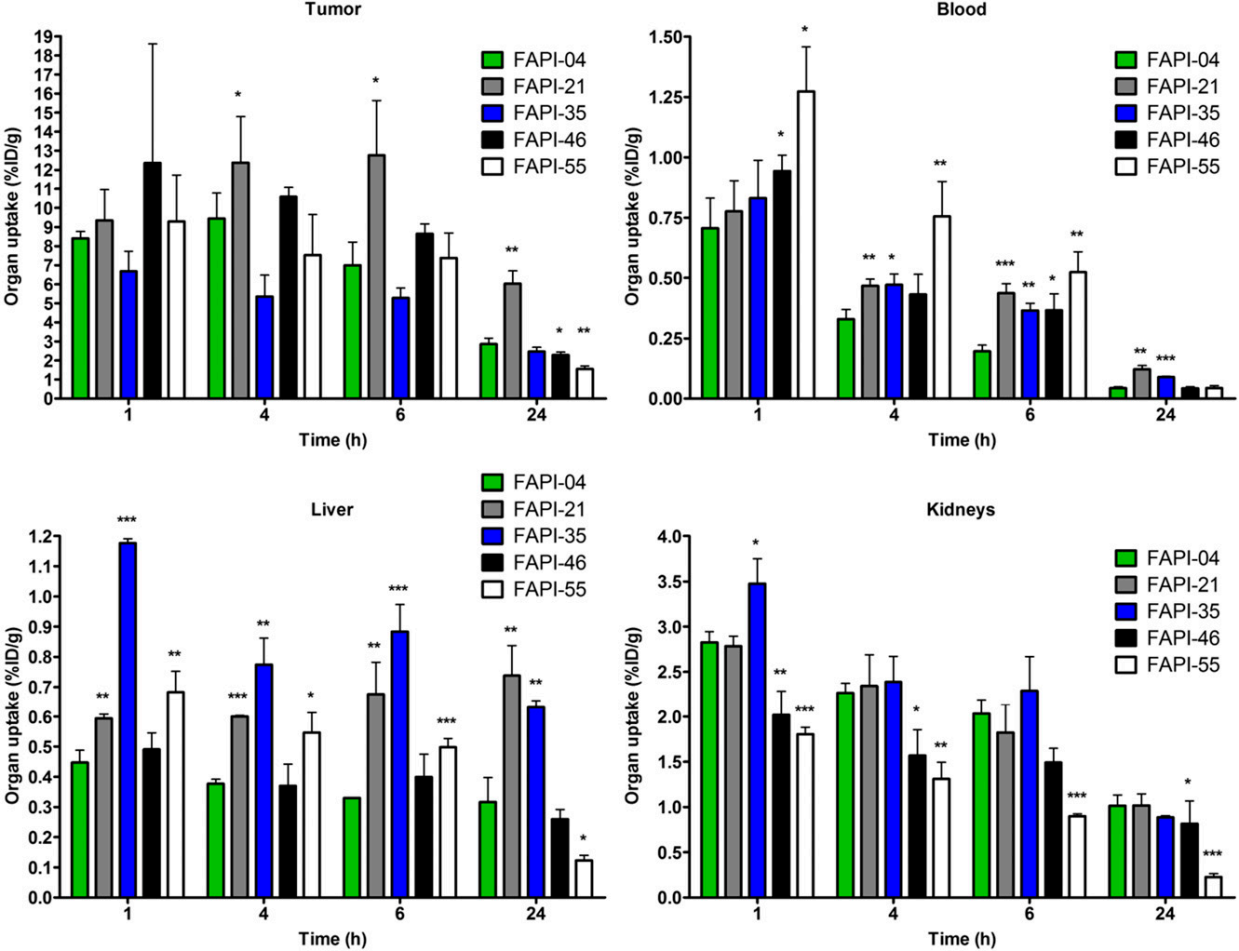
Organ uptake of ^177^Lu-labeled FAPI derivatives in HT-1080-FAP tumor–bearing mice (*n* = 3). *P < 0.05. **P < 0.01. ***P < 0.001.

**FIGURE 5.**
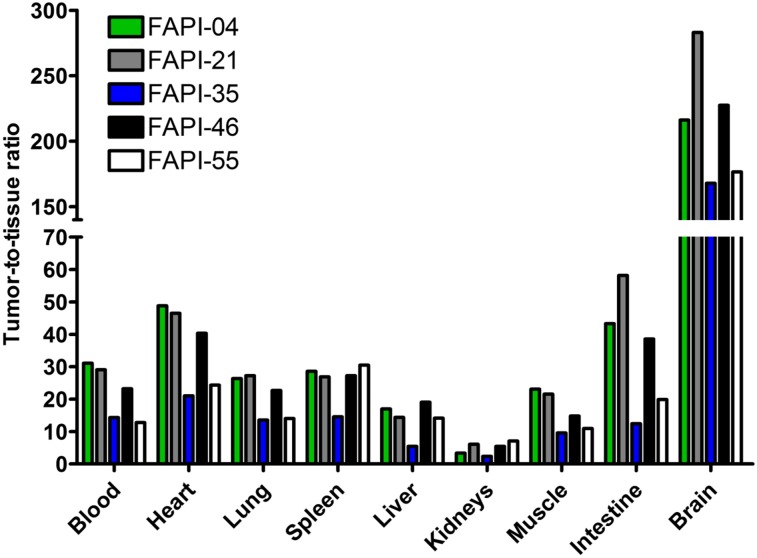
Tumor–to–normal-organ ratios (calculated from %ID/g 0–24 h after intravenous administration) of ^177^Lu-labeled FAPI derivatives in HT-1080-FAP tumor–bearing mice (*n* = 3).

On the basis of the observations in the imaging studies, FAPI-21, -35, -46, and -55 were selected for a more detailed characterization in biodistribution studies using ^177^Lu-labeled radiotracers. As shown in Figure 4, all compounds demonstrated robust tumor accumulation with overall low uptake into healthy tissue. Moderate radioactivity (1.8–3.5 %ID/g 1 h after injection) was measured only in the kidneys, because of the predominantly renal elimination of the radiotracers, with activity mostly in the renal calyx system. In comparison to FAPI-04, FAPI-21 and -46 demonstrated higher tumor accumulation 1 and 4 h after injection. Although all other compounds displayed their highest intratumoral radioactivity 1 h after injection, tumor uptake was increasing even from 1 to 4 h for FAPI-21. In addition, FAPI-21 revealed the highest tumor retention 24 h after injection (6.03 ± 0.68 %ID/g), followed by FAPI-35 (2.47 ± 0.23 %ID/g) and -46 (2.29 ± 0.16 %ID/g), featuring uptake rates similar to FAPI-04 (2.86 ± 0.31 %ID/g). Accordingly, 64% of the maximum tumor activity was still present 24 h after injection for FAPI-21, followed by FAPI-35 (37%), FAPI-46, and FAPI-55 (almost 20% each). In comparison to FAPI-04, radioactivity levels in the blood were equal or marginally higher at all specified times, except for FAPI-55, which demonstrated the highest blood activities of all compounds up to 6 h after injection. However, blood activity was decreasing steadily, reaching values similar to FAPI-04 after 24 h. All derivatives demonstrated an increased liver uptake as compared with FAPI-04, except for FAPI-46, which displayed comparable activities up to 6 h after injection but narrowed to lower levels in the course of 24 h. The renal activity of the compounds was comparable for FAPI-04, -21, and -35 but significantly reduced for FAPI-46 and -55 at all specified times. A comparison of AUCs, determined from the time–activity curves from 1 to 24 h after injection, revealed the highest overall tumor uptake to be for FAPI-21, followed by FAPI-46 (Table 2). A calculation of tumor-to-organ ratios, based on the overall AUCs, evinced a general improvement in pharmacokinetics for FAPI-21 and -46 and no considerable change for any of the other radiotracers, except for FAPI-35 (Fig. 5, Supplemental Table 3). Notably, FAPI-46 displayed substantially improved ratios of tumor to liver, kidney, and brain uptake.

**TABLE 2:** Tumor Uptake Rates of ^177^Lu-Labeled FAPI Derivatives and Calculated AUCs

Derivative	1 h	4 h	6 h	24 h	AUC 1−24 h
FAPI-04	8.40 ± 0.36	9.44 ± 1.33	7.00 ± 1.20	2.86 ± 0.31	7,915
FAPI-21	9.35 ± 1.62	12.38 ± 2.42	12.77 ± 2.88	6.03 ± 0.68	13,613
FAPI-35	6.68 ± 1.06	5.35 ± 1.13	5.29 ± 0.51	2.47 ± 0.23	5,902
FAPI-46	12.35 ± 6.25	10.60 ± 0.49	8.64 ± 0.52	2.29 ± 0.16	9,126
FAPI-55	9.30 ± 2.43	7.53 ± 2.13	7.37 ± 1.32	1.55 ± 0.16	7,289

Data are mean %ID/g ± SD (*n* = 3).

### Strong Accumulation of FAPI-21 and -46 in Various Tumors in Humans

**FIGURE 6.**
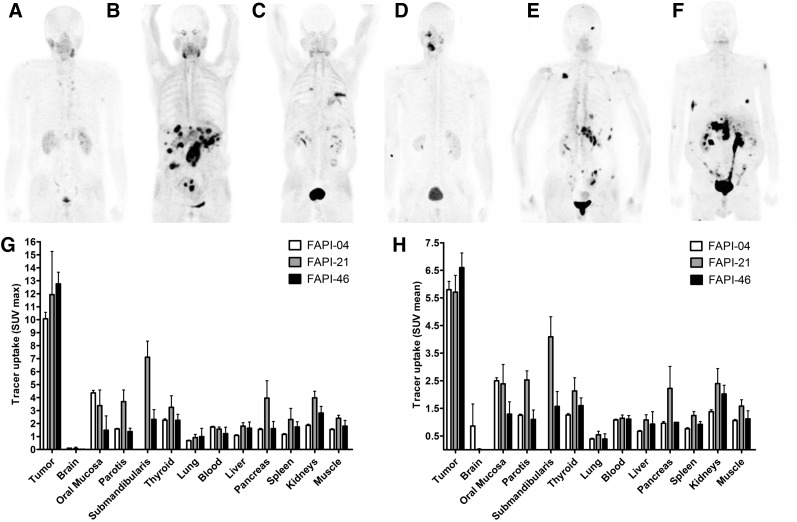
Whole-body PET/CT imaging of tumor patients. (A–F) Maximum-intensity projections 1 h after intravenous administration of ^68^Ga-labeled FAPI-21 (A–C) and FAPI-46 (D–F). (G and H) Maximum (G) and mean (H) tracer uptake of ^68^Ga-labeled FAPI-21 and -46 in tumor and healthy organs as compared with FAPI-04 (*n* = 2–25) (Supplemental Table 4).

Whole-body PET/CT scans were performed 10 min, 1 h, and 3 h after intravenous administration of ^68^Ga‐FAPI‐21 or -46 in patients with metastasized mucoepidermoid, oropharynx, ovarian, and colorectal carcinoma. Both radiotracers rapidly accumulated in the primary tumors and the metastases, with an SUV_max_ of 11.9 ± 3.33 for FAPI-21 and 12.76 ± 0.90 for FAPI-46 1 h after administration (Fig. 6). Additionally, tracer uptake into normal tissue was low. The radioactivity was cleared steadily from the bloodstream and excreted predominantly via the kidneys, resulting in high-contrast images. Interestingly, FAPI-21 demonstrated an increased accumulation in the oral mucosa (SUV_max_, 3.38 ± 1.20, vs. 1.49 ± 1.10 for FAPI-46), the thyroid (3.25 ± 0.89 vs. 2.25 ± 0.46), and the salivary glands (parotis, 3.69 ± 0.89 vs. 1.38 ± 0.26; submandibularis, 7.11 ± 1.24 vs. 2.32 ± 0.75).

### Demonstration of Higher Tumor Uptake with FAPI-46 Than with FAPI-04

In comparison to the lead compound FAPI-04, FAPI-46 displayed higher tumor uptake and comparable activities in healthy tissue (Figs. 6G and 6H). Notably, tumor accumulation rates highly depend on the tumor type. As shown in Table 3, tumor activity of the radiotracers remained relatively constant up to 3 h after injection in colorectal, ovarian, oropharyngeal, and pancreatic carcinoma, whereas a continuous decrease was observed in breast carcinoma. In contrast, tumor accumulation in 1 patient with carcinoma of unknown primary was even increasing from 1 to 3 h after administration (Table 3).

**TABLE 3:** Tumor Uptake at 10, 60, and 180 Minutes After Administration of ^68^Ga-Labeled FAPI-04, -21, and -46 to Cancer Patients

Patient with…	FAPI-04		FAPI-21			FAPI-46		
	60 min	180 min	10 min	60 min	180 min	10 min	60 min	180 min
Colorectal ca.	3 (4.77 ± 4.27)	3 (3.67 ± 3.41)						
Colorectal ca.	8 (5.20 ± 0.73)	8 (4.39 ± 1.19)						
Mammary ca	6 (3.98 ± 0.80)	6 (3.40 ± 0.78)						
Pancreatic ca.	3 (2.90 ± 0.70)	3 (2.90 ± 0.78)						
Ovarian ca.			3 (6.41 ± 1.23)	3 (7.48 ± 1.51)	3 (7.42 ± 1.71)			
Ovarian ca.			4 (5.27 ± 1.79)	4 (5.36 ± 1.46)	4 (5.10 ± 1.12)			
Colorectal ca.			2 (5.47 ± 1.83)	2 (4.52 ± 1.22)	2 (3.32 ± 1.06)			
Colorectal ca.						4 (6.85 ± 1.96)	4 (7.23 ± 2.06)	4 (6.40 ± 1.64)
Mammary ca.						7 (7.73 ± 1.86)	7 (5.97 ± 0.84)	7 (4.44 ± 0.96)
Oropharyngeal ca.						2 (6.01 ± 0.82)	2 (6.77 ± 0.65)	2 (6.31 ± 0.25)
CUP						4 (5.41 ± 2.76)	4 (6.45 ± 4.15)	4 (6.93 ± 4.42)

ca. = cancer; CUP = carcinoma of unknown primary.

Data are number of tumor lesions per patient followed by SUV_mean_ ± SD in parentheses.

## DISCUSSION

The focus of this work was to enhance tumor retention of FAP-targeting radiotracers while simultaneously retaining the excellent imaging contrast of FAPI-02 and FAPI-04—that is, to develop an optimized theranostic tracer. On this account, 15 novel derivatives were selected and compared with the currently used FAPI-04 with regard to target binding and pharmacokinetic profile. The first approach toward derivatization was the alteration of lipophilicity by variations of the linker region, which was chosen for 9 derivatives, mainly bicyclic analogs of the original piperazine moiety. The second approach aimed for modification of the chemistry used for DOTA/linker attachment at the quinoline moiety. It is based on the effect of the nitrogen atom in the quinoline-4-carboxamide moiety, which accounts for a more than 60-fold reduction in the half-maximal inhibitory concentration compared with an isosteric 1-naphtylcarboxamide–based inhibitor (14). The rationale was to improve target binding and physicochemical properties by fine tuning of the electron density at the quinoline moiety by different substituents, which results, for example, in a modified proton acceptor capability. Therefore, the initial ether oxygen was replaced by methylene, sulfur, amino, and methylamino moieties. With the intention of achieving synergistic effects, 2 compounds combining both approaches were additionally synthesized.

A first analysis of the binding properties in vitro revealed similar or improved FAP binding of all tested derivatives after 1 and 4 h of incubation as compared with FAPI-04. Although most of the compounds demonstrated higher binding values after 24 h, 4 radiotracers were eliminated significantly faster from FAP-expressing cells. Although the modifications of the piperazine moiety (e.g., the methylene-bridged diaminobicycloheptane of FAPI-21) or the linker region (e.g., insertion of the methylamino group for FAPI-46) had no significant influence on the half-maximal inhibitory concentrations (Supplemental Fig. 3), strong effects on in vitro efflux kinetics were observed (Supplemental Fig. 2). After incubation for 4 h, only 25% of the initial activity of FAPI-04 remained within the cells. In contrast, both FAPI-21 and FAPI-46 were eliminated significantly more slowly from the FAP-expressing cells, with 74% and 65%, respectively, of the initial activity being still detectable after 4 h. Although the less degradation-susceptible DOTA-diazabicycloheptane bond may explain the slower excretion of FAPI-21 than of FAPI-04, the longer retention of FAPI-46, which has the same DOTA-piperazine framework as FAPI-04, indicates that the elimination of the cell-bound FAPI tracers is an interaction of multiple processes not yet resolved.

On the basis of the in vitro results, 10 of the initial 15 compounds were selected for PET imaging in FAP-positive tumor–bearing mice. Of these, 7 derivatives demonstrated higher tumor uptake values than FAPI-04, headed by FAPI-21, -36, -46, and -55. FAP-specific binding in vivo was verified for FAPI-21 and -46 by competition experiments, demonstrating a complete block of tumor accumulation by unlabeled compound (Supplemental Fig. 6). Except for FAPI-35 and -46, all compounds demonstrated significantly higher activities in muscle tissue, resulting in decreased image contrast. Prolonged systemic circulation, possibly caused by increased albumin binding, was observed for FAPI-36, resulting in unfavorable tumor-to-blood ratios and poorer image contrast. Moreover, increased blood activities might promote myelotoxic effects and are, therefore, not desirable. FAPI-55, which showed the highest tumor uptake of all compounds, also displayed an increased activity in the liver due to higher lipophilicity. Regarding tumor accumulation, similar results were obtained in a biodistribution study in which FAPI-21, -46, and -55 revealed higher tumor uptake rates than FAPI-04. However, slightly distinct observations were made with regard to the liver and renal activity of the compounds as a consequence of altered elimination. Because excretory processes are strongly time-dependent, the pharmacokinetic profile within the first 2 h after injection appears different from the activities measured at later times up to 24 h after compound administration. Although FAPI-55 displayed robust hepatic uptake at early times, liver activity after 24 h was significantly decreased. At the same time, renal accumulation was comparatively low, suggesting a predominantly hepatobiliary elimination of the radiotracer. In general, only marginal liver and kidney uptake was observed for all novel derivatives, except for FAPI-35, indicating a rapid body clearance, thus legitimizing diagnostic clinical use.

On the basis of the overall improved tumor–to–normal-tissue ratios, FAPI-21 and -46 were chosen for a first diagnostic application in cancer patients. Both compounds demonstrated robust tumor uptake and overall low background activity. Yet, FAPI-21 displayed increased uptake in the oral mucosa, thyroid, parotid, and submandibular glands for reasons not known yet. This observation, however, represents a major limitation regarding potential therapeutic application of the tracers. Although the preclinical data suggested better performance for FAPI-21 than for FAPI-46, especially with regard to increased tumor uptake, FAPI-46 proved to be more suitable as a theranostic agent in clinical imaging studies because of its lower uptake in normal organs. This observation highlights the diverse nature of human xenotransplants used in mice as compared with tumor metastases in human patients. This may impede a direct translation of results from experimental studies into clinical practice. Unlike in cancer patients, xenograft tumors in mice evolve from a rather homogeneous cell population and are characterized by a relatively consistent protein expression. In the animal models used for our experiments, genetically modified FAP-expressing tumor cells were applied, representing a rather artificial tumor model as compared with the clinical situation. Herein, the PET signal is generated by the accumulation of the radiotracers in CAFs evolved from a multitude of different precursor cells and therefore characterized by different protein expression levels. In addition, the highest tracer uptake in numerous animal models is often observed in defined tumor areas adjacent to blood vessels, which are well supplied with blood. This placement allows a rapid accumulation of the radiotracers but a rapid efflux at the same time. In contrast, human tumors form complex, heterogeneous structures in which perfusion and expression of the target protein may vary significantly. The amount and distribution of FAP-expressing CAFs, as well as the number of FAP molecules per cell, may differ, resulting in different pharmacokinetic profiles for the radiotracers in different tumor types. We observed differences in behavior in different types of tumors in this limited cohort of patients: a constant intracellular activity in colorectal, ovarian, oropharyngeal, and pancreatic carcinoma; a continuous decrease in breast carcinoma; and an increasing tracer accumulation in a single patient with carcinoma of unknown primary (Table 3). A possible explanation might be the heterogeneous origin of CAFs, which may develop from resident fibroblasts, bone marrow–derived mesenchymal stem cells, endothelial cells, epithelial cells, and even adipocytes (15–17). Because of their differences in origin, these CAFs possibly display different proteomes with a strong variation or even lack of FAP expression. Whether this finding of varying kinetics can be extrapolated to these different tumor entities in general has to be determined in a larger number of patients. This type of study can be expected to reveal important information with respect to the indication for a FAPI-based endoradiotherapy. Tumors with a longer retention of the tracer may respond better than tumors with a fast elimination of the radiopharmaceutical.

## CONCLUSION

Based on the lead compound FAPI-04, which is characterized by rapid uptake into FAP-positive tumors followed by considerable elimination of the tracer, a series of derivatives was successfully developed. Notably, the modification of the linker region between the quinoline moiety and the chelator resulted in an increased tumor uptake and improved pharmacokinetic properties for the resulting amino derivatives, which represent a novel class of radiotracers. Especially, FAPI-46 demonstrated improved tumor-to-organ ratios, resulting in an enhanced image contrast for PET imaging. Depending on the tumor type, tumor accumulation could be significantly prolonged by the tracer FAPI-46.

## DISCLOSURE

This work was funded in part by the Federal Ministry of Education and Research, grant 13N 13341. Uwe Haberkorn, Anastasia Loktev, Thomas Lindner, Clemens Kratochwil, Frederik Giesel, and Walter Mier are named in a patent application (EP 18155420.5) for quinolone-based FAP-targeting agents for imaging and therapy in nuclear medicine. No other potential conflict of interest relevant to this article was reported.

## KEY POINTS

**QUESTION:** FAPI variants were developed to improve tumor uptake and retention.
**PERTINENT FINDINGS:** Of 15 FAPI variants, FAPI-46 showed substantially improved tumor to blood, liver, muscle, and intestinal uptake. Transfer into clinical application revealed high uptake in different tumor entities as early as 10 min after tracer administration.
**IMPLICATIONS FOR PATIENT CARE:** FAPI-46 led to high-contrast images for improved diagnostic application and may be used as a theranostic molecule also for therapy.
