# Supplementary material for: Development of Fibroblast Activation Protein–Targeted Radiotracers with Improved Tumor Retention
Source: J Nucl Med. 2019 Oct;60(10):1421–9. doi: 10.2967/jnumed.118.224469 (PMC6785792; doi:10.2967/jnumed.118.224469)
Supplement: Supplementary file 1 [file jnm224469SupplementalData.pdf]

Chemistry

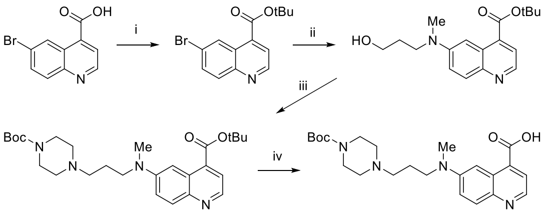

i) DCC, tBuOH, CuCl; ii) $HO(CH_2)_3NHMe$, $Pd_2(dba)_3$, BINAP, Cs_2CO_3; iii) 1) MsCl, NEt_3; 2) 1-Boc-piperazine, KI; iv) 1) TFA/TfOH; 2) Boc_2O, NEt_3.

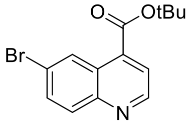

tert-butyl 6-bromoquinoline-4-carboxylate

98.3 mg (390 µmol) 6-bromoquinolie-4-carboxylic acid (raw) were suspended in 5 mL tetrahydrofuran and 25.0 µL (18.3 mg; 181 µmol) triethylamine and added to *O*-tert-butyl-*N*,*N'*-dicyclohexylisourea (prepared the day before from neat 426 mg (2.07 mmol) dicyclohexylcarbodiimide, 173 mg (2.33 mmol) tert-butanol and 10.2 mg (103 µmol) copper(I)iodide). The mixture was heated to 50 °C over night. The mixture was filtered, solvents evaporated and the product isolated by HPLC. 49.7 mg (161 µmol; 41%) of the title compound were obtained after freeze drying.

LC-MS R_t 20.40 min, m/z 251.9642 [M-*t*Bu]$^+$

tert-butyl 6-(4-chlorobutyl)quinoline-4-carboxylate

6.12 mg (19.9 µmol) *tert*-butyl 6-bromoquinoline-4-carboxylate, 1.81 mg (1.98 µmol) $Pd_2(dba)_3$ and 1.85 mg (4.50 µmol) S-Phos were dissolved in 1 mL dry tetrahydrofurane under an inert atmosphere. 200 µL (100 µmol) of a 0.5 M 4-chlorobutyl-1-zinc bromide solution in tetrahydrofurane were added and the reaction was stirred over night. The reaction was quenched with 500 µL of saturated ammonium chloride solution, both phases evaporated and taken up in water acetonitrile 1:1. The mixture was filtered by an oasiss C18 light column before HPLC. 5.82 mg (18.2 µmol; 91%) were obtained after freeze drying.

LC-MS R_t 18.83 min, m/z 320.1393 [M+H]$^+$

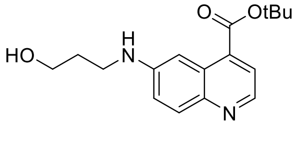

tert-butyl 6-(3-hydroxypropylamino)quinoline-4-carboxylate

6.14 mg (19.9 µmol) *tert*-butyl 6-bromoquinoline-4-carboxylate, 2.56 mg (4.11 µmol) BINAP, 1.61 mg (1.76 µmol) $Pd_2(dba)_3$ and 37.0 (113 µmol) cesium carbonate were dissolved in 1 mL toluene and 5.00 µL (4.95 mg; 65.9 µmol) 1,3-propanolamine were added. The mixture was stirred at 90 °C over night before solvents were removed, the residue suspended in water/acetonitrile 1:1 and filtered before HPLC-purification. 4.41 mg (14.6 µmol; 73%) of the title compound were obtained after freeze drying.

LC-MS R_t 12.95 min, m/z 303.1685 $[M+H]^+$

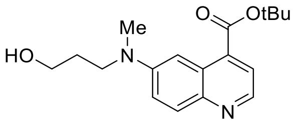

tert-butyl 6-(3-hydroxypropylmethylamino)quinoline-4-carboxylate

99.14 mg (322 µmol) *tert*-butyl 6-bromoquinoline-4-carboxylate, 12.26 mg (19.7 µmol) BINAP, 7.46 mg (8.14 µmol) $Pd_2(dba)_3$ and 212.85 mg (653 µmol) cesium carbonate were dissolved in 3 mL toluene and 64.0 µL (58.9 mg; 660 µmol) *N*-methyl-1,3-propanolamine were added. The mixture was stirred at 90 °C over night before solvents were removed, the residue suspended in water/acetonitrile 1:1 and filtered before HPLC-purification. 62.8 mg (199 µmol; 62%) of the title compound were obtained after freeze drying.

LC-MS R_t 13.41 min, m/z 261.1213 $[M\text{-}t\text{Bu}+H]^+$

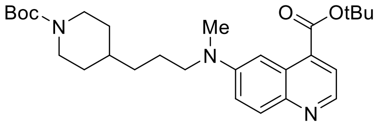

tert-butyl 6-(3-(1-Boc-piperidin-4-yl)propyl-1-(methyl)amino)quinoline-4-carboxylate

3.12 mg (6.45 µmol; 29%) were obtained following the previous method.

LC-MS R_t 19.04 min, m/z 484.3124 $[M+H]^+$

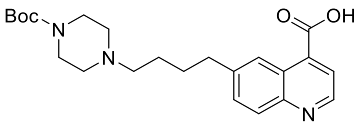

6-(4-(4-*tert*-butoxypiperazin-1-yl)butyl)quinoline-4-carboxylic acid

4.75 mg (14.8 µmol) *tert*-butyl 6-(4-chlorobutyl)quinoline-4-carboxylate were dissolved in 200 µL trifluoroacetic acid (with 2.5% water) and shaken for 180 minutes. 1 mL dichloromethane was added and the solvents removed in vacuo, which was repeated three times. 500 µL dimethylformamide, 35.2 mg (108 µmol) cesium carbonate, 52.41 mg (282 µmol) 1-Boc-piperazine and 7.22 mg (43.5 µmol) potassium iodide were added to the residue. The mixture was shaken at 60 °C over night and purified by HPLC. 3.02 mg (7.29 µmol; 49%) were obtained after freeze drying.

LC-MS R_t 10.78 min, m/z 414.2364 $[M+H]^+$

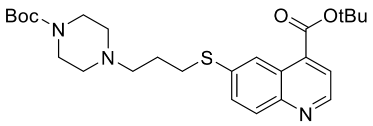

tert-butyl 6-(3-4-*tert*-butoxycarbonylpiperazin-1-ylpropyl-1-thio)quinoline-4-carboxylate

6.35 mg (20.6 µmol) *tert*-butyl 6-bromoquinoline-4-carboxylate, 2.95 mg (4.74 µmol) BINAP, 1.49 mg (1.53 µmol) $Pd_2(dba)_3$ and 36.1 (111 µmol) cesium carbonate were dissolved in 2 mL toluene and 14.78 mg (48.9 µmol) 3-(4-Boc-piperazin-1-yl)-1-(acetylthio)propane were added. The mixture was stirred at 90 °C over night before solvents were removed, the residue suspended in water/acetonitrile 1:1 and filtered before HPLC-purification. 7.82 mg (16.0 µmol; 78%) of the title compound were obtained after freeze drying.

LC-MS R_t 15.96 min, m/z 488.2550 $[M+H]^+$

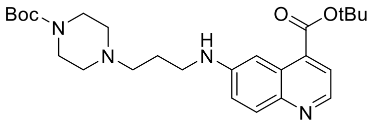

tert-butyl 6-(3-(4-Boc-piperazin-1-yl)propyl-1-amino)quinoline-4-carboxylate

2.39 mg (7.91 µmol) *tert*-butyl 6-(3-hydroxypropylamino)quinoline-4-carboxylate were dissolved in 1 mL dichloromethane and 5.5 µL (4.02 mg; 39.8 µmol) DIPEA. 1.00 µL (1.48 mg; 12.9 µmol) methanesulfonyl chloride were added and the mixture shaken for 60 min. 53.42 mg (28.7 µmol) 1-Boc-piperazine were added before volatiles were removed. 500 µL dimethylformamide and 19.22 mg (116 µmol) potassium iodide were added to the residue. The mixture was shaken at 60 °C for 120 minutes before the product was isolated by HPLC. 3.34 mg (7.09 µmol; 90%) of the title compound were obtained after freeze drying.

LC-MS R_t 13.54 min, m/z 471.2942 $[M+H]^+$

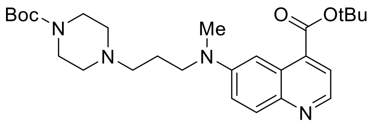

tert-butyl 6-(3-(4-Boc-piperazin-1-yl)propyl-1-(methyl)amino)quinoline-4-carboxylate

62.8 mg (199 µmol) *tert*-butyl 6-(3-hydroxypropylmethylamino)quinoline-4-carboxylate were dissolved in 5 mL dichloromethane and 90.0 µL (66.6 mg; 659 µmol) triethylamine. 20.0 µL (29.6 mg; 258 µmol) methanesulfonyl chloride were added at 0 °C and the mixture reacted for 60 min. 194.6 mg (1.05 mmol) 1-Boc-piperazine were added before volatiles were removed. 500 µL dimethylformamide and 47.4 mg (286 µmol) potassium iodide were added to the residue. The mixture was shaken at 60 °C for 120 minutes before the product was isolated by HPLC. 81.05 mg (167 µmol; 84%) of the title compound were obtained after freeze drying.

LC-MS R_t 13.99 min, m/z 485.3086 $[M+H]^+$

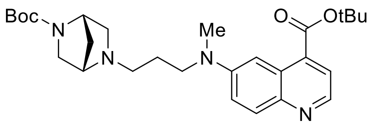

tert-butyl 6-(3-((1*S*,4*S*)-5-Boc-2,5-diazabicyclo[2.2.1]heptan-2-yl)propyl-1-(methyl)amino)quinoline-4-carboxylate

4.05 mg (8.15 µmol; 64%) were obtained following the previous protocol, while the reaction with (1*S*,4*S*)-2-Boc-2,5-diazabicyclo[2.2.1]heptan was carried out at 40 °C over night.

LC-MS R_t 13.79 min, m/z 497.3078 $[M+H]^+$

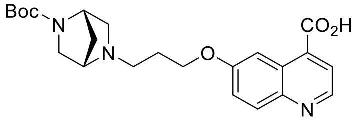

6-(3-((1*S*,4*S*)-5-(*tert*-butoxycarbonyl)-2,5-diazabicyclo[2.2.1]heptan-2-yl)propoxy)quinoline-4-carboxylic acid

3.42 mg (12.9 µmol) of 6-(3-chloro-1-propoxy)quinoline-4-carboxylic acid, 13.3 mg (66.9 µmol) (1*S*,4*S*)-2-Boc-2,5-diazabicyclo[2.2.1]heptan and 18.4 mg (111 µmol) potassium iodide were dissolved in 250 µL DMF. The reaction was shaken at 60 °C over night. The resulting suspension was diluted with 750 µL water before the product was purified by HPLC. After freeze drying 6.46 mg (11.9 µmol; 92%) of the product were obtained as the corresponding TFA-salt.

LC-MS R_t 10.66 min, m/z 450.1768 $[M+Na]^+$

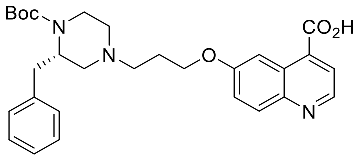

(*S*)-6-(3-(3-benzyl-4-(*tert*-butoxycarbonyl)piperazin-1-yl)propoxy)quinoline-4-carboxylic acid

10.1 mg (16.3 µmol; 85%) were obtained following the previous method.

LC-MS R_t 13.48 min, m/z 506.2388 $[M+H]^+$

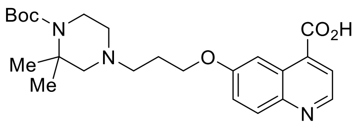

6-(3-(4-(*tert*-butoxycarbonyl)-3,3-dimethylpiperazin-1-yl)propoxy)quinoline-4-carboxylic acid

5.15 mg (9.23 µmol; 46%) were obtained following the previous method.

LC-MS R_t 11.85 min, m/z 444.2260 $[M+H]^+$

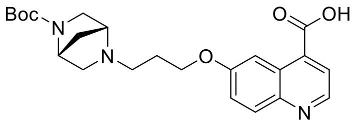

6-(3-((1*R*,4*R*)-5-(*tert*-butoxycarbonyl)-2,5-diazabicyclo[2.2.1]heptan-2-yl)propoxy)quinoline-4-carboxylic acid

2.13 mg (4.98 µmol; 93%) were obtained following the previous method.

LC-MS R_t 10.59 min, m/z 428.2147 $[M+H]^+$

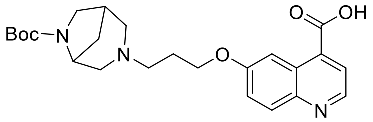

6-(3-(6-(*tert*-butoxycarbonyl)-3,6-diazabicyclo[2.2.1]heptan-2-yl)propoxy)quinoline-4-carboxylic acid

1.59 mg (3.60 µmol; 36%) were obtained following the previous method.

LC-MS R_t 10.92 min, m/z 442.2325 $[M+H]^+$

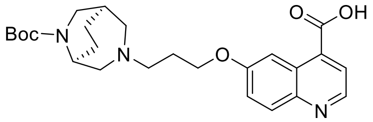

6-(3-((1*R*,5*S*)-6-(*tert*-butoxycarbonyl)-3,6-diazabicyclo[2.2.2]nonan-2-yl)propoxy)quinoline-4-carboxylic acid

3.52 mg (7.73 µmol; 75%) were obtained following the previous method.

LC-MS R_t 11.28 min, m/z 456.2478 $[M+H]^+$

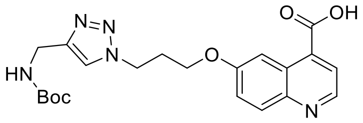

6-(3-(4-(*tert*-butoxycarbonylaminomethyl)-1*H*-1,2,3-triazol-1-yl)propoxy)quinoline-4-carboxylic acid

1.09 mg (4.01 µmol) 6-(3-azidopropoxy)quinoline-4-carboxylic acid were dissolved in 200 µL water and 1 µL (0.86 mg; 15.6 µmol) propargylamine. 1 µL saturated copper(II)acetate in water (0.36 µmol) were added and the solution was heated to 95 °C. The mixture was cooled to room temperature before 50 µL acetonitrile, 4.68 mg (21.5 µmol) di-*tert*-butyl dicarbonate and 5 µL (3.65 mg; 36.1 µmol) triethylamine were added. The product was isolated by HPLC after 60 min. 1.05 mg (2.45 µmol; 61%) of the title compound were obtained after freeze drying.

LC-MS R_t 12.20 min, m/z 428.1907 $[M+Na]^+$

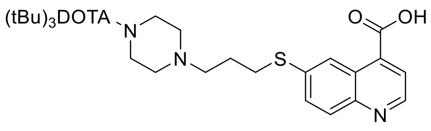

6-(3-(4-(tris-*t*BuDOTA)piperazin-1-yl)propyl-1-thio)quinoline-4-carboxylic acid

4.73 mg (9.69 µmol) *tert*-butyl 6-(3-4-*tert*-butoxycarbonylpiperazin-1-ylpropyl-1-thio)quinoline-4-carboxylate were deprotected with 200 µL trifluoroacetic acid containing 2.5% triethylsilane and 2.5% water for 120 min. The volatiles were removed by coevaporation with dichloromethane (3×1 mL) and the residue dissolved with 100 µL dimethylformamide and 5.50 µL (4.07 mg; 31.6 µmol). Meanwhile 6.89 mg (12.0 µmol) DOTA-tris(*t*Bu)ester and 4.69 mg (12.4 µmol) HBTU were reacted for 10 min in 150 µL dimethylformamide before addition to the deprotected quinoline solution. Finally 10.0 µL (7.40 mg; 57.4 µmol) DIPEA were added and the mixture reacted for 120 min. 6.59 mg (7.44 µmol; 77%) of the title compound were obtained after HPLC-purification and freeze-drying.

LC-MS R_t 13.00 min, m/z 908.4873 $[M+Na]^+$

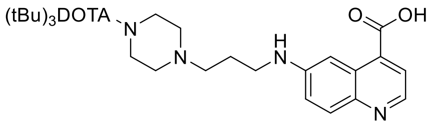

6-(3-(4-(tris-*t*BuDOTA)piperazin-1-yl)propyl-1-amino)quinoline-4-carboxylic acid

1.11 mg (1.28 µmol; 26%) were obtained following the previous protocol.

LC-MS R_t 12.06 min, m/z 891.5258 $[M+Na]^+$

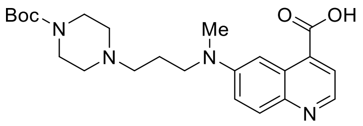

6-(3-(4-Boc-piperazin-1-yl)propyl-1-(methyl)amino)quinoline-4-carboxylic acid

100.12 mg (206 µmol) *tert*-butyl 6-(3-(4-Boc-piperazin-1-yl)propyl-1-(methyl)amino)quinoline-4-carboxylate were treated with 900 µL trifluoroacetic acid, 25 µL triisopropylsilane, 25 µL water and 50 µL trifluoromethanesulfonic acid for 60 min. The deprotected compound was precipitated with diethyl ether, dried and reacted with 60.83 mg (279 µmol) di-*tert*-butyldicarbonate and 50.0 µL (36.5 mg; 361 µmol) triethylamine in 1 mL dimethylformamide for another 60 min. 55.42 mg (129 µmol; 65% over 2 steps) were obtained after HPLC-purification and freeze-drying.

LC-MS R_t 10.52 min, m/z 429.2463 $[M+H]^+$

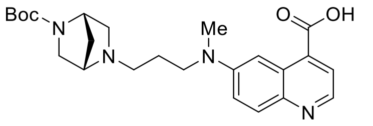

6-(3-((1*S*,4*S*)-5-Boc-2,5-diazabicyclo[2.2.1]heptan-2-yl)propyl-1-(methyl)amino)quinoline-4-carboxylic acid

2.48 mg (5.62 µmol; 88%) were obtained following the previous method.

LC-MS R_t 10.52 min, m/z 441.2464 $[M+H]^+$

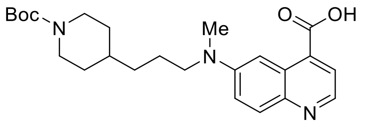

6-(3-(1-Boc-piperidin-4-yl)propyl-1-(methyl)amino)quinoline-4-carboxylic acid

1.57 mg (3.67 µmol; 57%) were obtained following the previous method.

LC-MS R_t 15.51 min, m/z 450.2324 $[M+Na]^+$

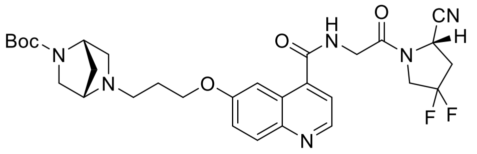

(*S*)-*N*-(2-(2-cyano-4,4-difluoropyrrolidin-1-yl)-2-oxoethyl)-6-(3-((1*S*,4*S*)-5-*tert*-butoxycarbonyl-2,5-diazabicyclo[2.2.1]heptan-2-yl)propoxy)quinoline-4-carboxamide

2.06 mg (5.44 µmol) HBTU in 50 µL DMF were added to a solution of 1.86 mg (4.35 µmol) 6-(3-(4-*tert*-butoxycarbonylpiperazin-1-yl)-1-propoxy)quinoline-4-carboxylic acid, 1.65 mg (12.2 µmol) HOBt and 2.50 µL (1.85 mg; 12.3 µmol) DIPEA in 50 µL DMF. After 15 min 2.26 mg (6.26 µmol) (*S*)-1-(2-aminoacetyl)pyrrolidine-2-carbonitrile 4-methylbenzenesulfonate in 50 µL DMF were added. The reaction was quenched with 500 µL water and purified by HPLC. Freeze drying provided 1.96 mg (3.27 µmol; 75%) of the title compound.

LC-MS R_t 12.41 min, m/z 599.2476 $[M+H]^+$

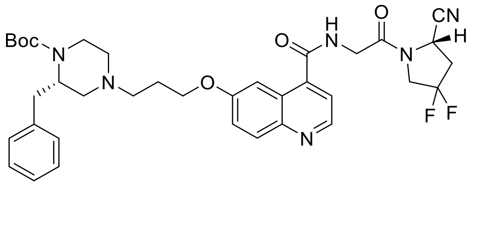

(*S*)-*N*-(2-(2-cyano-4,4-difluoropyrrolidin-1-yl)-2-oxoethyl)-6-(3-(3-(*S*)-benzyl-4-*tert*-butoxycarbonylpiperazin-1-yl)propoxy)quinoline-4-carboxamide

2.54 mg (3.75 µmol; 47%) were obtained following the previous method.

LC-MS R_t 14.83 min, m/z 677.2905 $[M+H]^+$

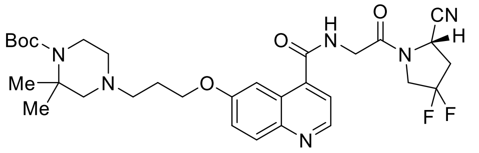

(*S*)-*N*-(2-(2-cyano-4,4-difluoropyrrolidin-1-yl)-2-oxoethyl)-6-(3-(4-(*tert*-butoxycarbonyl)-3,3-dimethylpiperazin-1-yl)propoxy)quinoline-4-carboxamide

2.33 mg (3.79 µmol; 84%) were obtained following the previous method.

LC-MS R_t 13.65 min, m/z 637.2586 $[M+Na]^+$

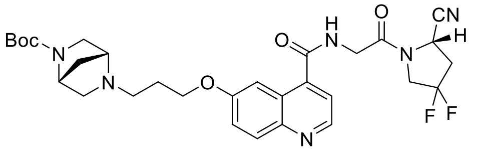

(*S*)-*N*-(2-(2-cyano-4,4-difluoropyrrolidin-1-yl)-2-oxoethyl)-6-(3-((1*R*,4*R*)-5-(*tert*-butoxycarbonyl)-2,5-diazabicyclo[2.2.1]heptan-2-yl)propoxy)quinoline-4-carboxamide

1.58 mg (2.64 µmol; 87%) were obtained following the previous method.

LC-MS R_t 12.44 min, m/z 599.2747 $[M+H]^+$

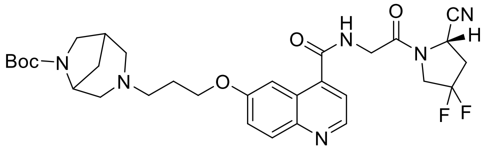

(*S*)-*N*-(2-(2-cyano-4,4-difluoropyrrolidin-1-yl)-2-oxoethyl)-6-(3-(6-(*tert*-butoxycarbonyl)-3,6-diazabicyclo[3.2.1]octan-3-yl)propoxy)quinoline-4-carboxamide

0.57 mg (0.93 µmol; 46%) were obtained following the previous method.

LC-MS R_t 12.59 min, m/z 613.2918 $[M+H]^+$

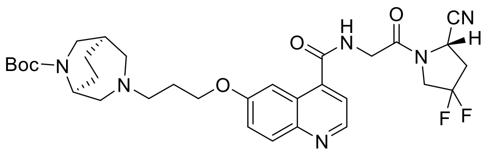

(*S*)-*N*-(2-(2-cyano-4,4-difluoropyrrolidin-1-yl)-2-oxoethyl)-6-(3-((1*R*,5*S*)-6-(*tert*-butoxycarbonyl)-3,6-diazabicyclo[3.2.2]nonan-3-yl)propoxy)quinoline-4-carboxamide

1.22 mg (1.95 µmol; 41%) were obtained following the previous method.

LC-MS R_t 13.08 min, m/z 627.3075 $[M+H]^+$

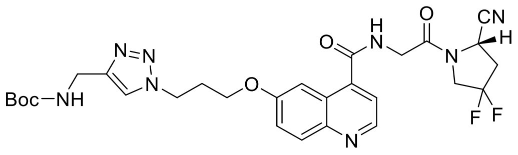

(*S*)-*N*-(2-(2-cyano-4,4-difluoropyrrolidin-1-yl)-2-oxoethyl)-6-(3-(4-(*tert*-butoxycarbonylaminomethyl)-1*H*-1,2,3-triazol-1-yl)propoxy)quinoline-4-carboxamide

0.68 mg (1.14 µmol; 78%) were obtained following the previous method.

LC-MS R_t 13.66 min, m/z 599.2506 $[M+H]^+$

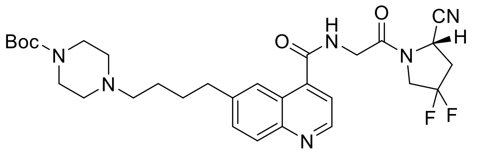

(*S*)-*N*-(2-(2-cyano-4,4-difluoropyrrolidin-1-yl)-2-oxoethyl)-6-(4-(4-*tert*-butoxycarbonylpiperazin-1-yl)butyl)quinoline-4-carboxamide

3.02 mg (7.29 µmol; 49%) were obtained following the previous method.

LC-MS R_t 13.18 min, m/z 585.2961 $[M+H]^+$

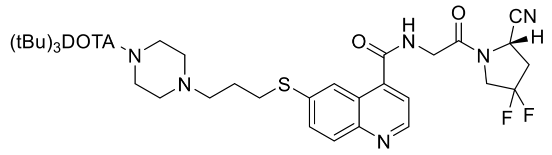

N-(2-(2-cyano-4,4-difluoropyrrolidin-1-yl)-2-oxoethyl)-6-(3-(4-Boc-piperazin-1-yl)propyl-1-thio)quinoline-4-carboxamide

4.91 mg (4.65 µmol; 81%) were obtained following the previous method.

LC-MS R_t 13.78 min, m/z 1079.5466 $[M+Na]^+$

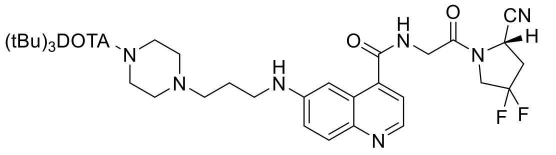

N-(2-(2-cyano-4,4-difluoropyrrolidin-1-yl)-2-oxoethyl)-6-(3-(4-Boc-piperazin-1-yl)propyl-1-amino)quinoline-4-carboxamide

0.74 mg (0.71 µmol; 79%) were obtained following the previous method.

LC-MS R_t 12.89 min, m/z 1062.5860 $[M+H]^+$

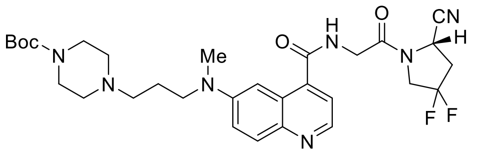

N-(2-(2-cyano-4,4-difluoropyrrolidin-1-yl)-2-oxoethyl)-6-(3-(4-Boc-piperazin-1-yl)propyl-1-(methyl)amino)quinoline-4-carboxamide

0.60 mg (1.0 µmol; 37%) were obtained following the previous method.

LC-MS R_t 12.66 min, m/z 600.3057 $[M+H]^+$

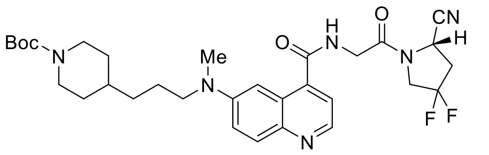

(*S*)-*N*-(2-(2-cyano-4,4-difluoropyrrolidin-1-yl)-2-oxoethyl)-6-(3-(1-Boc-piperidin-4-yl)propyl-1-amino)quinoline-4-carboxamide

51.94 mg (86.6 µmol; 82%) were obtained following the previous method.

LC-MS R_t 16.28 min, m/z 621.2915 $[M+Na]^+$

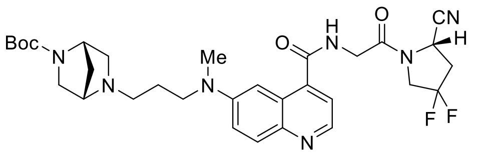

(*S*)-*N*-(2-(2-cyano-4,4-difluoropyrrolidin-1-yl)-2-oxoethyl)-6-(3-((1*S*,4*S*)-5-*tert*-butoxycarbonyl-2,5-diazabicyclo[2.2.1]heptan-2-yl)propyl-1-(methyl)amino)quinoline-4-carboxamide

1.89 mg (3.09 µmol; 78%) were obtained following the previous method.

LC-MS R_t 12.35 min, m/z 612.3062 $[M+H]^+$

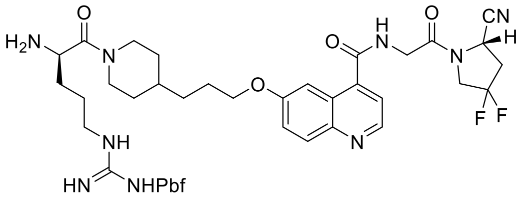

(*S*)-*N*-(2-(2-cyano-4,4-difluoropyrrolidin-1-yl)-2-oxoethyl)-6-(3-(1-D-Arg(Pbf)-piperidin-4-yl)-1-propoxy)quinoline-4-carboxamide

1.11 mg (1.89 µmol) of (*S*)-*N*-(2-(2-cyanopyrrolidin-1-yl)-2-oxoethyl)-6-(3-(1-*tert*-butoxycarbonyl-piperidin-4-yl)-1-propoxy)quinoline-4-carboxamide and 1.87 mg (9.79 µmol) 4-methylbenzene-sulfonic acid monohydrate were dissolved in 50 µL acetonitrile. After 60 min the volatiles removed. To the residue was dissolved in 50 µL dimethylformamide and 2.50 µL (1.85 mg; 14.3 µmol) DIPEA and added to a solution of 2.62 mg (4.04 µmol) Fmoc-D-Arg(Pbf)-OH, 0.61 mg (4.52 µmol) HOBt, 1.70 mg (4.49 µmol) HBTU and 2.50 µL (1.85 mg; 14.3 µmol) DIPEA in 50 µL dimethylformamide. After 60 min 22.2 µL (22.4 mg; 257 µmol) morpholin were added and the product was isolated by HPLC after further 90 min. 1.55 mg (1.73 µmol; 92%) of the title compound were obtained after freeze drying.

LC-MS R_t 14.38 min, m/z 447.7088 $[M+2H]^{2+}$

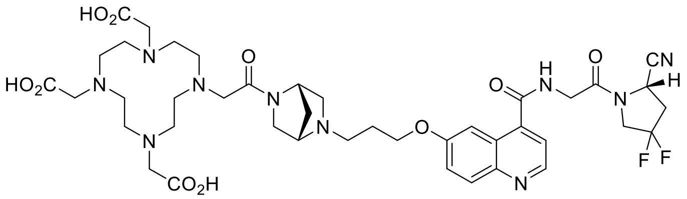

FAPI-21

0.84 mg (1.41 mmol) (*S*)-*N*-(2-(2-cyano-4,4-difluoropyrrolidin-1-yl)-2-oxoethyl)-6-(3-((1*S*,4*S*)-5-Boc-2,5-diazabicyclo[2.2.1]heptan-2-yl)propoxy)quinoline-4-carboxamide were dissolved in 30 µL acetonitrile and 60 µL trifluoroacetic acid. The reaction was shaken for 15 min before volatiles were removed and the residue precipitated by diethyl ether. after centrifugation the solid was taken up in 190 µL dimethylformamide and 5.00 µL (3.65 mg; 36.1 µmol) triethylamine before 1.64 mg (3.12 µmol) of DOTA-*p*-nitrophenol ester were added. The reaction mixture was diluted with 1 mL water and purified by HPLC after shaking for two hours. 0.84 mg (0.95 µmol; 67%) were obtained after freeze drying.

LC-MS R_t 8.90 min, m/z 885.3605 $[M+H]^+$

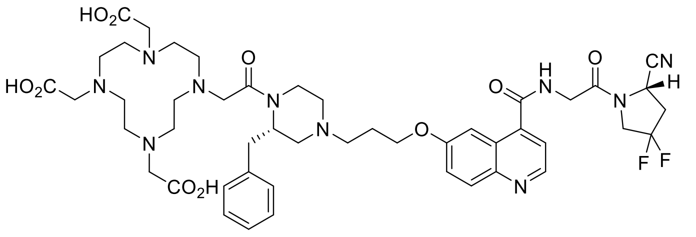

FAPI-22

0.92 mg (0.96 µmol; 67%) were obtained following the previous protocol.

LC-MS R_t 10.18 min, m/z 985.3843 $[M+Na]^+$

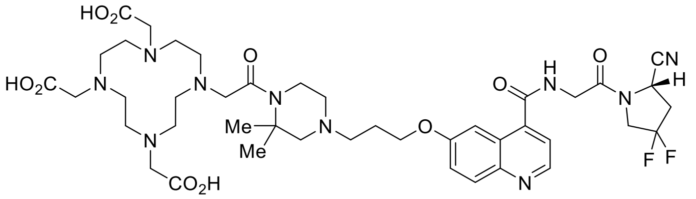

FAPI-23

0.21 mg (0.23 µmol; 14%) were obtained following the previous protocol.

LC-MS R_t 9.13 min, m/z 923.3715 $[M+H]^+$

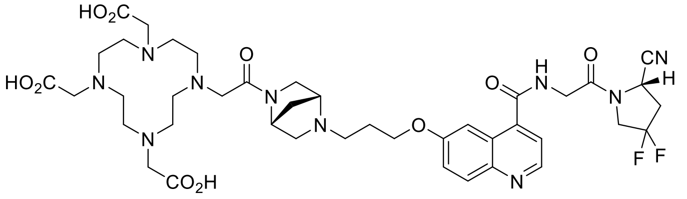

FAPI-31

0.21 mg (0.24 µmol; 14%) were obtained following the previous protocol.

LC-MS R_t 8.92 min, m/z 443.2058 $[M+2H]^{2+}$

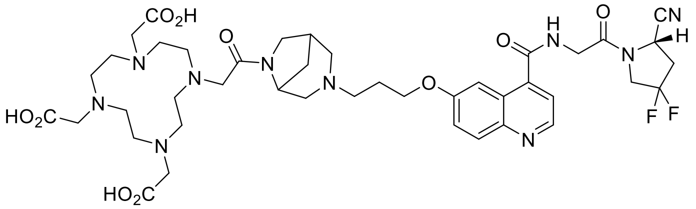

FAPI-35

0.26 mg (0.29 µmol; 71%) were obtained following the previous protocol.

LC-MS R_t 9.26 min, m/z 450.2127 $[M+2H]^{2+}$

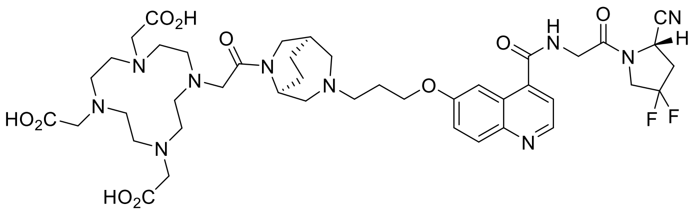

FAPI-36

0.49 mg (0.54 µmol; 96%) were obtained following the previous protocol.

LC-MS R_t 9.36 min, m/z 457.2205 $[M+2H]^{2+}$

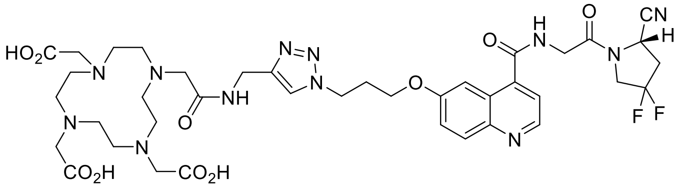

FAPI-37

0.69 mg (0.78 µmol; quant.) were obtained following the previous protocol.

LC-MS R_t 9.66 min, m/z 443.1920 $[M+2H]^{2+}$

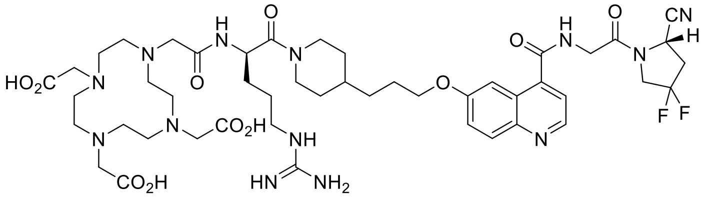

FAPI-38

0.97 mg (0.94 µmol; 78%) were obtained following the previous protocol.

LC-MS R_t 10.46 min, m/z 514.7572 $[M+2H]^{2+}$

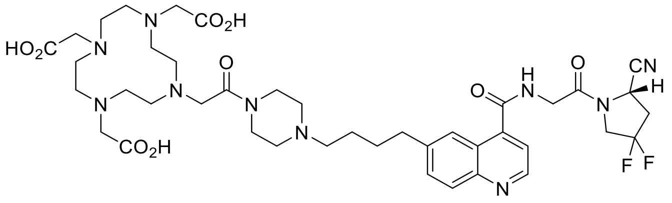

FAPI-39

0.91 mg (1.04 µmol; 71%) were obtained following the previous protocol.

LC-MS R_t 9.19 min, m/z 871.4217 $[M+H]^+$

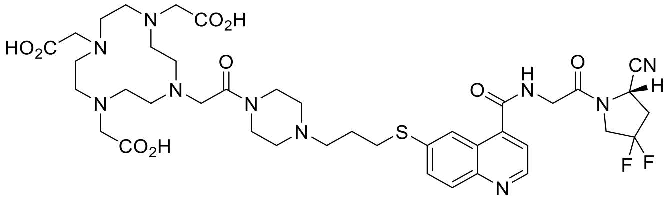

FAPI-40

2.23 mg (2.51 µmol; 69%) were obtained after deprotection with 2.5% trifluorometahesulfonic acid in trifluoroacetic acid/acetonitrile 8:2 for 5 min and HPLC-purification/freeze-drying.

LC-MS R_t 9.63 min, m/z 889.3783 $[M+H]^+$

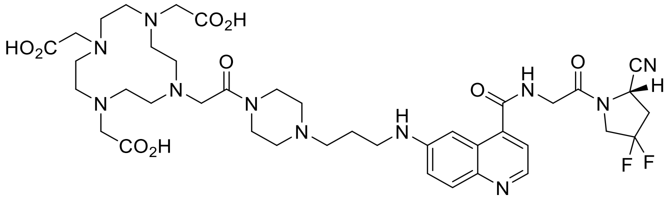

FAPI-41

0.24 mg (0.28 µmol; 74%) were obtained after deprotection with 2.5% trifluorometahesulfonic acid in trifluoroacetic acid/acetonitrile 8:2 for 5 min and HPLC-purification/freeze-drying.

LC-MS R_t 8.56 min, m/z 436.7125 $[M+2H]^{2+}$

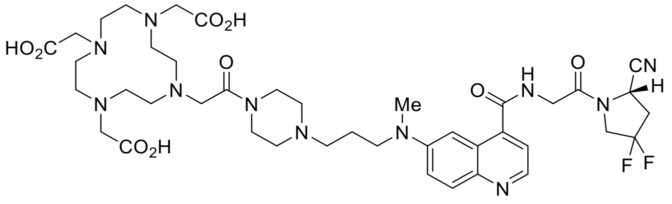

FAPI-46

39.21 mg (44.3 µmol; 85%) were obtained following the procedure for FAPI-21.

LC-MS R_t 9.03 min, m/z 443.7196 $[M+2H]^{2+}$

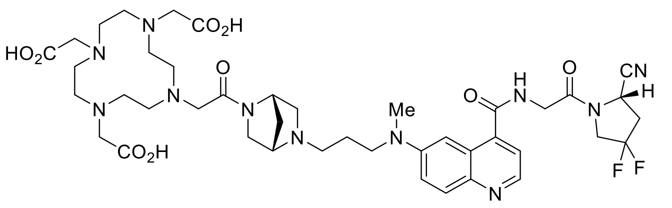

FAPI-53

0.81 mg (0.91 µmol; 41%) were obtained following the previous protocol.

LC-MS R_t 9.09 min, m/z 449.7194 $[M+2H]^{2+}$

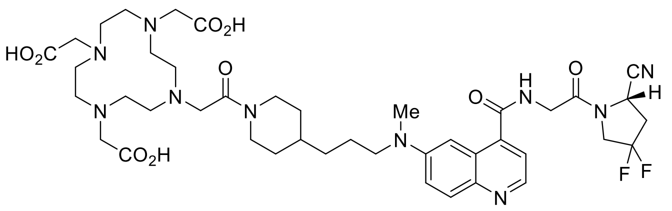

FAPI-55

0.27 mg (0.31 µmol; 63%) were obtained following the previous protocol.

LC-MS R_t 10.78 min, m/z 443.2211 $[M+2H]^{2+}$

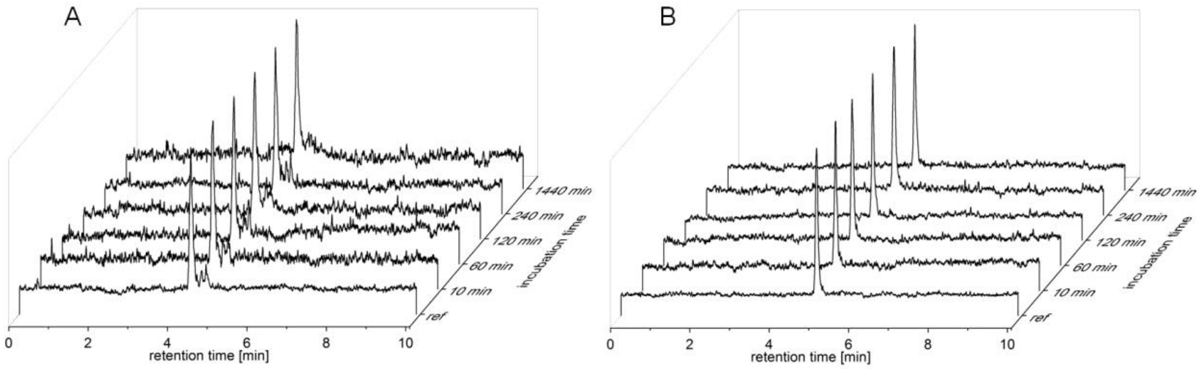

Supplemental Fig. 1. Stability in human serum of A) FAPI-21 and B) FAPI-46.

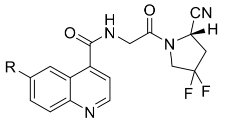

Compound	R-	Compound	R-
FAPI-04	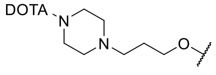	FAPI-37	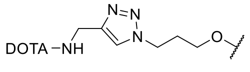
FAPI-20	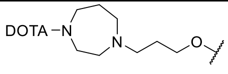	FAPI-38	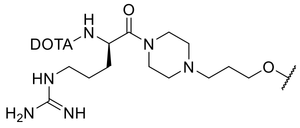
FAPI-21	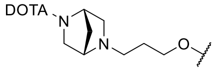	FAPI-39	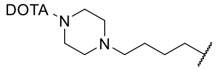
FAPI-22	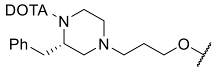	FAPI-40	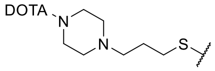
FAPI-23	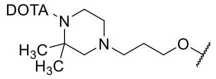	FAPI-41	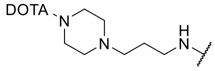
FAPI-31	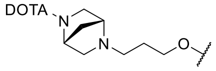	FAPI-46	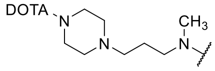
FAPI-35	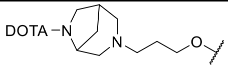	FAPI-53	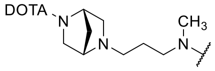
FAPI-36	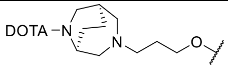	FAPI-55	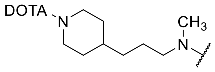

Supplemental Table 1. Chemical structure of the novel FAPI derivatives

In vitro results

	1 h	4 h	24 h
**FAPI-04**	94,44	94,80	97,09
**FAPI-20**	97,79	97,75	96,50
**FAPI-21**	98,04	97,52	97,20
**FAPI-22**	97,39	96,87	95,16
**FAPI-23**	95,64	96,45	96,61
**FAPI-31**	96,35	nd	86,04
**FAPI-35**	97,03	97,75	95,33
**FAPI-36**	96,82	96,71	90,13
**FAPI-37**	97,96	97,40	93,72
**FAPI-38**	94,77	94,59	63,14
**FAPI-39**	94,61	94,45	90,90
**FAPI-40**	95,31	94,92	89,38
**FAPI-41**	96,81	97,27	83,91
**FAPI-46**	97,18	97,75	92,03
**FAPI-53**	93,74	92,97	88,23
**FAPI-55**	94,60	95,29	94,88

Supplemental Table 2. Percentage of internalized fraction of selected FAPI derivatives in HT-1080-FAP cells after incubation for 1, 4 and 24 h; nd: *not determined*.

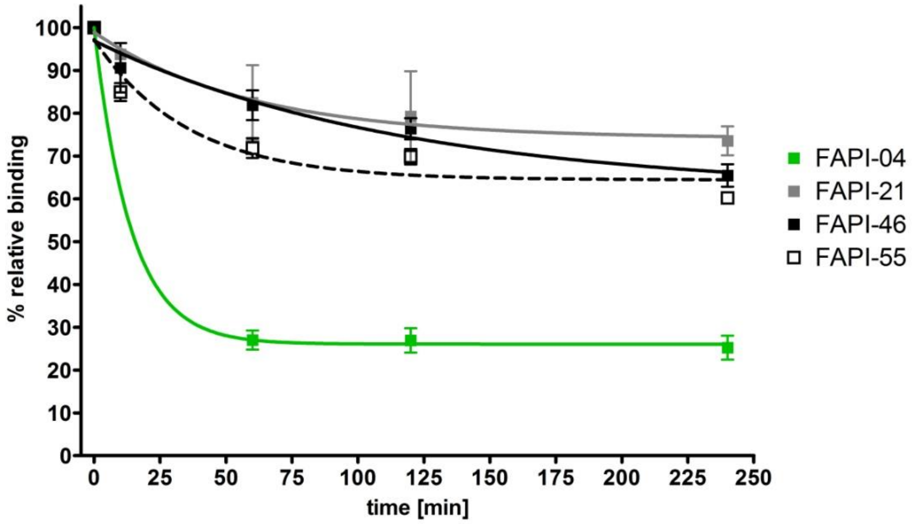

Supplemental Fig. 2. Efflux kinetics of selected FAPI derivatives after incubation of HT-1080-FAP cells with radiolabeled compound for 60 min and consequent incubation with nonradioactive medium for 1 to 4 hours.

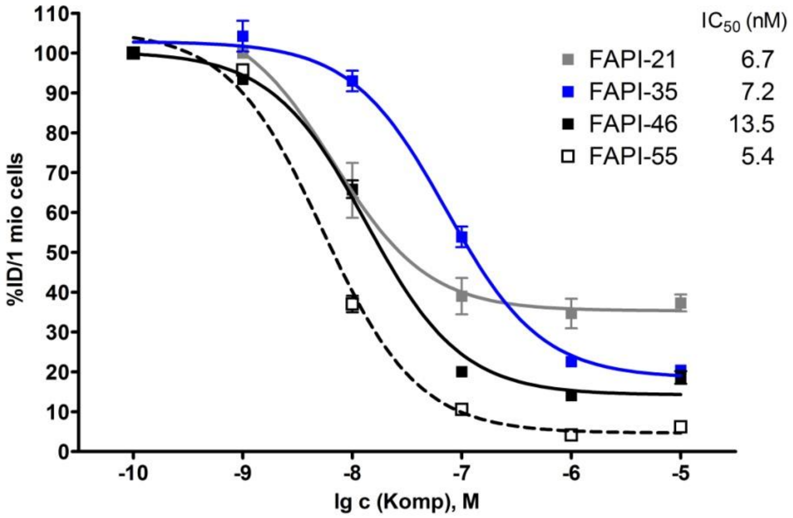

Supplemental Fig. 3. Competitive binding of selected FAPI derivatives to HT-1080-FAP cells after adding increasing concentrations of unlabeled compound.

Small animal imaging

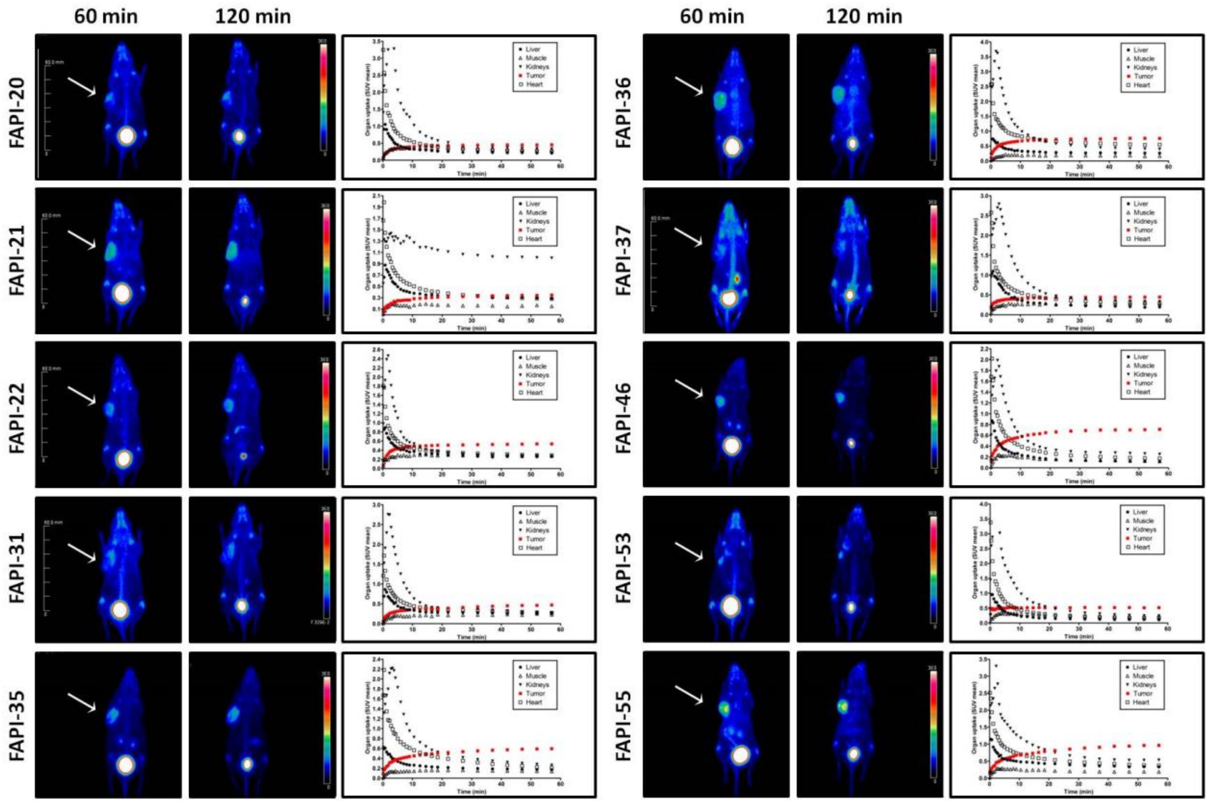

Supplemental Fig. 4. PET imaging of selected FAPI derivatives in HT-1080-FAP tumor bearing mice. Maximum intensity projections (MIP) 60 and 120 min after intravenous injection of ^{68}Ga-labeled compound (tumor indicated by the arrow); time-activity curves up to 60 min after injection.

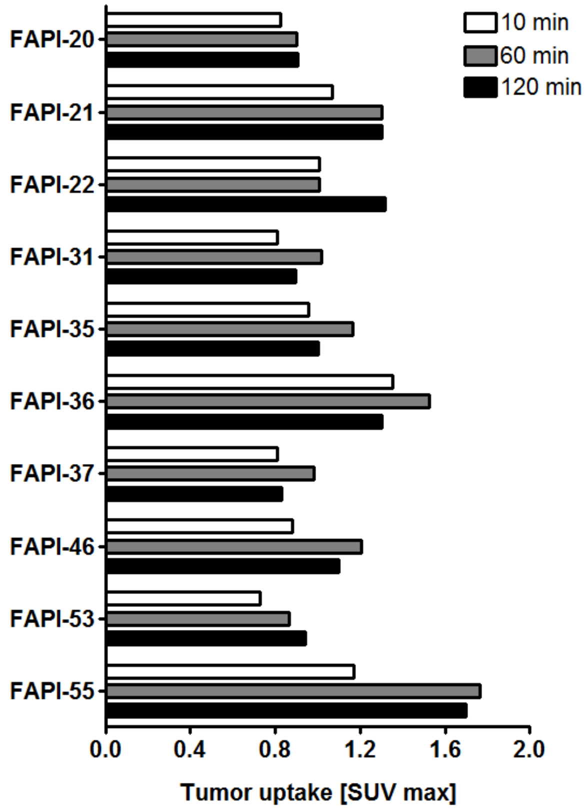

Supplemental Fig. 5. Maximum tumor uptake of ^{68}Ga-labeled FAPI derivatives up to 120 min after intravenous administration, determined by small animal PET imaging.

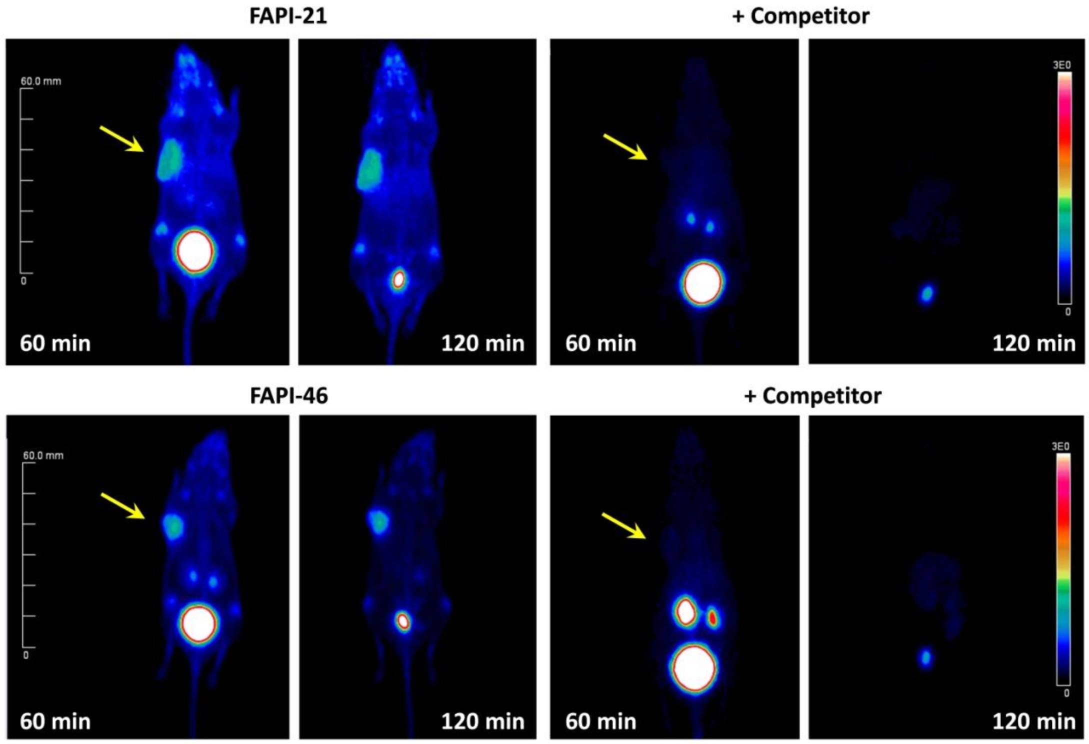

Supplemental Fig. 6. PET imaging of FAPI-21 and -46 in HT-1080-FAP tumor bearing mice. Maximum intensity projections (MIP) 60 and 120 min after intravenous injection of ^{68}Ga-labeled compound (tumor indicated by the arrow) with and without simultaneous administration of unlabeled compound as competitor; n=1.

	Blood	Heart	Lung	Spleen	Liver	Kidney	Muscle	Intestine	Brain
**FAPI-04**	31.10	48.80	26.33	28.58	17.00	3.35	23.10	43.28	216.26
**FAPI-21**	29.09	46.52	27.24	26.86	14.37	6.07	21.55	58.15	282.99
**FAPI-35**	14.32	21.00	13.56	14.55	5.40	2.34	9.55	12.41	167.90
**FAPI-46**	23.19	40.33	22.66	27.24	19.05	5.40	14.76	38.54	227.41
**FAPI-55**	12.77	24.35	14.02	30.52	14.15	7.09	10.91	19.87	176.60

Supplemental Table 3. Tumor-to-normal tissue ratios (calculated from %ID/g values 0-24 h after intravenous administration) of ^{177}Lu-labeled FAPI derivatives in HT-1080-FAP tumor bearing mice.

	FAPI-04			FAPI-21			FAPI-46		
	**max**	**mean**	**n**	**max**	**mean**	**n**	**max**	**mean**	**n**
**Tumor**	10.07 ± 0.50	5.8 ± 0.3	25	11.93 ± 3.33	5.71 ± 0.61	3	12.76 ± 0.90	6.60 ± 0.53	4
**Brain**	0.10 ± 0.01	0.86 ± 0.8	25	0.07 ± 0.11	0.01 ± 0.03	4	0.02 ± 0.02	0.00 ± 0.00	4
**Oral Mucosa**	4.36 ± 0.19	2.50 ± 0.11	25	3.38 ± 1.20	2.39 ± 0.70	4	1.49 ± 1.10	1.29 ± 0.45	4
**Parotis**	1.58 ± 0.05	1.25 ± 0.04	25	3.69 ± 0.89	2.53 ± 0.33	4	1.38 ± 0.26	1.10 ± 0.34	4
**Submandibularis**	not determined			7.11 ± 1.24	4.09 ± 0.73	4	2.32 ± 0.75	1.57 ± 0.54	4
**Thyroid**	2.26 ± 0.11	1.26 ± 0.05	25	3.25 ± 0.89	2.13 ± 0.48	4	2.25 ± 0.46	1.60 ± 0.28	4
**Lung**	0.68 ± 0.04	0.39 ± 0.03	25	0.92 ± 0.25	0.54 ± 0.13	4	0.99 ± 0.64	0.39 ± 0.19	4
**Blood**	1.73 ± 0.06	1.08 ± 0.03	25	1.57 ± 0.16	1.14 ± 0.12	4	1.22 ± 0.50	1.11 ± 0.13	4
**Liver**	1.09 ± 0.05	0.67 ± 0.03	25	1.80 ± 0.26	1.08 ± 0.19	4	1.64 ± 0.48	0.93 ± 0.45	4
**Pancreas**	1.55 ± 0.08	0.96 ± 0.06	25	3.96 ± 1.34	2.22 ± 0.80	2	1.61 ± 0.55	0.99 ± 0.01	3
**Spleen**	1.17 ± 0.06	0.76 ± 0.04	25	2.31 ± 0.86	1.24 ± 0.14	3	1.74 ± 0.40	0.92 ± 0.10	4
**Kidneys**	1.86 ± 0.09	1.38 ± 0.07	25	3.98 ± 0.51	2.40 ± 0.54	4	2.81 ± 0.51	2.02 ± 0.32	4
**Muscle**	1.54 ± 0.06	1.06 ± 0.04	25	2.41 ± 0.23	1.58 ± 0.23	4	1.80 ± 0.44	1.12 ± 0.29	4

Supplemental Table 4. SUV max and mean values ± standard deviation 1 h after administration of ^{68}Ga-labeled FAPI-04, -21 and -46 to cancer patients; *n: number of patients*. The FAPI-04 data in 25 patients were taken from Giesel, F. *et al.* FAPI-PET/CT: biodistribution and preliminary dosimetry estimate of two DOTA-containing FAP-targeting agents in patients with various cancers. *Journal of nuclear medicine: official publication, Society of Nuclear Medicine*, doi:10.2967/jnumed.118.215913 (2018).
